# Swedish translation and cultural adaptation of the General Well-Being Schedule for patients with olfactory and hearing dysfunction

**DOI:** 10.3389/fpsyg.2026.1896914

**Published:** 2026-08-26

**Authors:** Sofie Henecke, Anja L. Winter, Evelina Thunell, Johan N. Lundström, Ylva Tiblom Ehrsson

**Affiliations:** 1 Department of Clinical Neuroscience, Karolinska Institutet, Stockholm, Sweden; 2 Department of Otorhinolaryngology, Karolinska University Hospital, Stockholm, Sweden; 3 Monell Chemical Senses Center, Philadelphia, PA, United States; 4 Department of Surgical Sciences, Section of Otorhinolaryngology and Head and Neck Surgery, Uppsala University, Uppsala, Sweden

**Keywords:** cultural adaptation, hearing, olfaction disorders, quality of life, translations

## Abstract

**Background:**

Quality of life (QoL) is largely determined by our psychological well-being, yet commonly used somatic QoL measures often emphasize physical symptoms. Consequently, psychological needs in individuals who lack physical comorbidities are often overlooked. The General Well-Being Schedule (GWB) is a validated tool for assessing well-being, without focusing on physical dysfunction or being disease specific. As sensory impairments are strongly linked to reduced QoL, a Swedish GWB could have substantial clinical value.

**Objective:**

To translate and culturally adapt the GWB into Swedish following the guidelines of the International Society for Health Economics and Outcomes Research (ISPOR), and to establish adequate content validity of the resulting instrument.

**Methods:**

The GWB was translated into Swedish from English by following the ISPOR guidelines. Forward and backward translations were performed. Both healthcare professionals and patients with olfactory or hearing disorders contributed their opinions on the translation through focus groups, interviews, and ratings. Based on their input, we calculated the Scale Content Validity Index (S-CVI) and the Item Content Validity Index (I-CVI), to measure the questionnaire’s overall content validity and the content validity of each item, respectively.

**Results:**

The focus groups and interviews resulted in several linguistic and cultural adjustments. Content validity was excellent for the questionnaire as a whole with an S-CVI of 0.91. Out of the 18 questions, 16 had an excellent I-CVI (mean = 0.94).

**Conclusion:**

The GWB was successfully translated and culturally adapted for use in Sweden, following the ISPOR guidelines. The excellent content validity of the translation supports the relevance of the translated instrument.

## 1 Introduction

Our life quality heavily depends on the proper functioning of our sensory systems (Henderson et al., 2025; Barker et al., 2017; Croy et al., 2014; Liu et al., 2021; Liu et al., 2022; Saniasiaya and Prepageran, 2021). At the same time, quality of life (QoL) is in large part determined by subjective and psychological well-being (Ring et al., 2007). This is demonstrated by the observation that in patient populations, some patients suffer considerably while others, with the same level of symptom severity, maintain a satisfactory life quality (Elwenspoek et al., 2024; Ayis et al., 2015).

Sensory dysfunction refers to an impairment in the reception, integration, or interpretation of sensory stimuli. The dysfunction can affect any of the sensory modalities, including hearing and olfaction, both of which are considered key sensorineural functions essential for daily functioning and healthy aging (Wongrakpanich et al., 2016). Hearing disorders can heavily affect the health-related quality of life (HRQoL) and its impact extends well beyond auditory function illustrated by its negative effects on psychological wellbeing, social participation, and occupational performance (Olusanya et al., 2014). Although sensory disorders collectively carry a well-documented burden on quality of life (QoL), the impact of olfactory dysfunction (OD) has historically been underestimated, in part because smell has not been regarded as a vital sense in the same way as vision or hearing. Recent research has nonetheless demonstrated that OD, whether manifesting as quantitative loss (anosmia, hyposmia) or qualitative distortion (parosmia, phantosmia), negatively affects several areas of daily life, including nutrition, social functioning, and psychological well-being (Oleszkiewicz et al., 2025). For a significant proportion of affected individuals, the psychological consequences are particularly pronounced, with elevated rates of depressive symptoms, anxiety, apathy, loneliness, and general psychological distress (Croy and Hummel, 2017). Despite this, in somatic healthcare, many QoL measures are to a large extent addressing physical well-being, such as pain or motor and mobility disorders (Pequeno et al., 2020). The problem of QoL-scale’s focus on physical ailments is of particular relevance for individuals with sensory impairments. Because these patients often lack comorbidities, they often score within normative range on these standard QoL instruments, and their ubiquitous subjective and psychological QoL issues are left unaddressed. To target this, several specific measurements have been developed to evaluate QoL in patients with sensory dysfunction. For patients with olfactory dysfunction, the Questionnaire of Olfactory Disorders (QOD) (Frasnelli and Hummel, 2005) and its derivative Questionnaire of Olfactory Disorders-Negative Statements (QOD-NS) are often used (Mattos et al., 2019). For hearing disorders, the Hearing Handicap Inventory for the Elderly (HHIE) (Weinstein et al., 1986) or the Health Utilities Index Mark III (HUI3) are most common (Yang et al., 2013). However, these, as well as many of the other QoL instruments predominantly used in somatic healthcare, are disease-specific which complicates comparisons across health conditions. Instruments assessing overall well-being, without an emphasis on specific conditions or physical complaints, are therefore important for many patient populations; specifically those with a sensory dysfunction.

The General Well-Being Schedule (GWB) is a widely used and validated short questionnaire designed to assess an individual’s subjective experience of their general psychological well-being during the past month, without a focus on physical ailments. The questionnaire was originally developed at the United States’ principal health statistics agency, The National Center for Health Statistics (NCHS) in 1970, (Fazio, 1977; Fish, 2011). It has since been used and validated in various large-scale populations with good results, showing reliability across different populations (Poston et al., 1998; Taylor et al., 2003; Nakayama et al., 2000; Leonardson et al., 2003). The GWB consists of 18 questions that explore different aspects of psychological well-being on six subscales: self-control, depression, anxiety, positive well-being, vitality, and general health. All questions refer to the past month and aim to provide a picture of the patient’s recent general psychological well-being. Fourteen of the questions use a six-point scale, while the remaining four have a 0–10 scale with opposing adjectives at each end. Item scores are summed to yield a total score ranging from 0 to 110, with higher scores indicating greater psychological well-being. Total scores are typically interpreted according to established cut-off points: scores of 73–110 reflect positive well-being, 61–72 indicate moderate distress, and scores below 61 suggest severe distress. These thresholds allow the GWB to function as a continuous measure of well-being but also as a screening tool for identifying individuals at risk of psychological distress (Fazio, 1977; Fish, 2011). Even though the questionnaire has been validated and widely used internationally, it has not been translated or culturally adapted for a Swedish setting.

Despite the strong connection between reduced QoL and sensory dysfunctions, there is no well-established Swedish tool to identify patients at risk. A culturally adapted Swedish version of the GWB has a potential high clinical impact as an aid to identify patients in need of psychological support. To this end, patients seeking treatment for either olfactory or hearing dysfunctions were used as a proxy for individuals with general sensory processing impairments. In Sweden, about 1 in 5 adults live with a hearing disorder. Hearing disorders, which can include both quantitative (hearing loss) and qualitative (tinnitus) aspects, are known to reduce physical, mental and social QoL (Henderson et al., 2025; Barker et al., 2017). Another sensory disorder that has become increasingly prevalent after the COVID-19 pandemic is olfactory dysfunction and similar to hearing disorders, an estimated 1 in 5 adults live with olfactory dysfunction in Sweden (Brämerson et al., 2004). As with hearing, olfactory disorders can be either quantitative (smell loss) or qualitative (smell distortion) but often present simultaneously (Cho, 2014). While the link between olfactory disorders and both depression and a reduced QoL was established long before the pandemic, the additional research that emerged as a result has further strengthened the support for this connection (Liu et al., 2021; Saniasiaya and Prepageran, 2021).

Many questionnaires used in healthcare are developed in English-speaking countries, and it is important to translate these into more languages to enable cross-country comparisons. However, for patient-reported outcome measures to be used across different cultural contexts, linguistic translation alone is insufficient; items must also undergo cultural adaptation to ensure that the conceptual meaning and content validity of the original instrument are preserved in the target population. When translating and adapting an instrument, it is important to follow a systematic and well-structured process to preserve the validity and reliability of the original instrument. A poorly executed translation risks introducing semantic, or conceptual, errors and inconsistencies between the source and target versions, which can compromise the instrument and undermine cross-cultural comparability of results. A rigorous, stepwise approach allows discrepancies between the source and target language versions to be systematically identified and resolved before the instrument is used in research or clinical practice (Beaton et al., 2000). Following internationally recognized guidelines also enhances the transparency, reproducibility, and credibility of the adaptation process, allowing other researchers to evaluate and replicate the methodology. A systematic translation process safeguards the scientific integrity of the adapted instrument, ensuring it measures the same underlying construct as the original and produces data that are both valid and meaningful within the new cultural and linguistic context (Wild et al., 2005). The International Society for Health Economics and Outcomes Research (ISPOR) has established the “Principles of Good Practice for the Translation and Cultural Adaptation Process for Patient-Reported Outcomes (PRO) Measures” to guide this procedure. This approach ensures a cultural adaptation to fit the target population (Wild et al., 2005). There are several methods to evaluate the quality of a translated measure. The Content Validity Index (CVI) provides a quantitative method for evaluating the extent to which adapted items are considered relevant, clear, and representative by a group of subject matter experts. In translation and cultural adaptation studies, the CVI serves as a systematic and transparent mechanism for ensuring that adapted items retain their intended meaning and remain appropriate for the target population. By incorporating expert judgment in a structured and quantifiable manner, the CVI complements the linguistic steps of the adaptation process and contributes to the overall evidence base for the validity of the translated instrument (Lynn, 1986; Polit and Beck, 2006).

## 2 Purpose

The main purpose of this study was to culturally adapt and translate the English GWB into Swedish, for patients with hearing and olfactory disorders, following the ISPOR guidelines, and to establish adequate content validity of the resulting instrument.

## 3 Methods

### 3.1 Ethical approval and informed consent

This study was performed in line with the principles of the Declaration of Helsinki and approved by the Swedish Ethical Review Authority (dnr 2024-00973-02). All participants received oral and written information, and their written consent was obtained.

### 3.2 ISPOR steps

To ensure a standardized method for the translation and cultural adaptation, we followed the guidelines of the International Society for Health Economics and Outcomes Research (ISPOR). These guidelines contain 10 steps, from preparation to final report (Wild et al., 2005).

#### 3.2.1 ISPOR step 1: preparation

Permission was obtained, via e-mail, from the NCHS to use and translate the GWB according to the ISPOR guidelines from English into Swedish. All group members working with the translation are native Swedish speakers with extensive experience in working and writing in English where several lived between 5 to 12 years in North America. One is an associate professor of nursing, one a professor of psychology, one an assistant professor of psychology and two are PhD candidates (one of them a registered nurse specializing in ear, nose and throat patients).

#### 3.2.2 ISPOR step 2–6: forward translation, back translation and harmonization

In step 2, two of the authors (SH and ET) made forward translations of the GWB from English into Swedish individually. In step 3, the two versions were discussed and compared in a Reconciliation workshop where all authors attended. A final version was agreed on, with the aim of capturing the original version while fitting into a Swedish context. In step 4, a back translation was made from Swedish into English by co-author ALW. In step 5, the back translation was reviewed to ensure that the new version maintained the sentiment of the original. In step 6, there was a harmonization of all the translations and the original version to identify any discrepancies between the versions. Consensus on a final Swedish version was reached after minor adjustments.

#### 3.2.3 ISPOR step 7: cognitive debriefing

The cognitive debriefing was performed in three phases: A, B, and C.

#### 3.2.4 Phase A: clinical focus groups

In Phase A, healthcare professionals working with the target patient populations were recruited to focus groups to test comprehensibility and phrasing. Healthcare professionals were involved as subject-matter experts to evaluate the clinical relevance, terminology, and contextual appropriateness of the items before they were tested with patients. All recruited healthcare professionals worked at the Ear, Nose, Throat and Hearing Clinic at the Karolinska University Hospital in Stockholm, Sweden. One focus group consisted of three professionals working with patients with hearing dysfunctions, and another consisted of three professionals working with patients with olfactory dysfunction (Table 1). Informed consent was obtained from all participants after receiving oral and written information about the study. The focus groups were recorded with the participants consent; the recordings were later erased after being analyzed. The discussions were moderated by author SH using open-ended question focusing on the comprehensibility, clarity and the cultural appropriateness of the questionnaire. Focus groups were followed up by a reconciliation meeting between authors SH and YTE, during which changes were made based on what emerged from both focus groups. These modifications aimed to improve comprehensibility and cultural relevance while preserving conceptual equivalence with the original instrument. The number of healthcare professionals included was aligned with the recommended numbers. The revised version was then used in phase B.

**Table 1:** Characteristics of participants.

Activity	Profession	Years in profession (median, range)	Years in field (median, range)
Focus group: hearing (*n* = 3)	2 audiologists	16 (7–18)	7 (3–18)
	1 physician (audiology specialist)		
Focus group: olfaction (*n* = 3)	2 nurses	11 (5–12)	3 (2–4)
	1 physician (rhinologist in olfaction)		
Survey: content validity (*n* = 10)	4 nurses	15 (2–35)	4 (1–32)
	3 physicians		
	2 audiologists		
	1 assistant nurse		

Numbers (*n*) are given.

#### 3.2.5 Phase B: patient cognitive debriefing interviews

In phase B, consistent with ISPOR recommendations regarding sample size for cognitive debriefing, four patients with hearing disorders and four patients with olfactory disorders, who had visited the clinic the previous week, were recruited from the Ear, Nose, Throat and Hearing Clinic at the Karolinska University Hospital in Stockholm, Sweden to individual interviews. Informed consent was obtained from all participants after receiving oral and written information about the study. The interviews were conducted by author SH, about the patients’ perspectives and thoughts on the questionnaire and its phrasing [5 female, 3 male, median age 53 years (min = 38, max = 67)]. To evaluate whether respondents understood the questionnaire and its items, in the way they were intended to be interpreted, the ISPOR methodology for cognitive interviews were followed (Patrick et al., 2011). A combination of think-aloud and probing techniques were used. In the think-aloud component, participants were asked to verbalize their thoughts spontaneously while reading and responding to each item, allowing the interviewer to observe how meaning was constructed in real time and to identify confusion, or misinterpretation as it occurred. Additional probes explored clarity, interpretation, relevance, comprehensibility, and cultural appropriateness. Any items identified as problematic were discussed within the research team and revised accordingly.

The changes were incorporated into a revised version of the questionnaire that was used for Phase C.

#### 3.2.6 Phase C: clinical assessment of content validity

In phase C, following patient cognitive debriefing, an additional content validity assessment was conducted with healthcare professionals to evaluate the relevance and applicability of each item in Swedish clinical practice. Ten healthcare professionals (Table 1) working with either hearing or olfactory dysfunction, that had not taken part in the focus groups, were recruited at the Ear, Nose, Throat and Hearing Clinic at the Karolinska University Hospital in Stockholm, Sweden. Informed consent was obtained from all participants after receiving oral and written information about the study. The healthcare professionals were asked to rate their perceived relevance of each question in the translated questionnaire using a Likert scale (1 = very irrelevant, 2 = irrelevant, 3 = relevant, and 4 = very relevant). In addition, two open-ended questions addressed aspects related to relevance and operational use. The survey was administered on paper, not online. As above, the number of healthcare professionals included was aligned with the recommended numbers.

#### 3.2.7 ISPOR step 8–10: review of cognitive debriefing results and finalization, proofreading and final report

In steps 8–10, authors SH and YE reviewed the result of the cognitive debriefing (step 7) and adapted the translation accordingly (step 8). The resulting version was then corrected by a professional proofreader (paid contributor) who had not been involved in the translation process (step 9). All steps of the translation and adaptation process were documented and summarized for publication in a peer-reviewed scientific journal, to make out the final report (step 10).

### 3.3 Analysis

The results of the focus group meetings (step 7, phase A) and interviews (step 7, phase B) were reviewed in reconciliation meetings between authors SH and YE. Changes to the questionnaire, suggested by the professionals and patients during Step 7, were discussed and implemented when deemed appropriate in improving the quality of the questionnaire without losing sentiment of the original version.

The experts’ rated relevance of the questions (step 7, phase C) was used to assess the item content validity index (I-CVI) of each question. The CVI is typically calculated at both the item level (I-CVI) and the scale level (S-CVI). The I-CVI was defined as the proportion of experts who rated the item with either 3 or 4 (indicating agreement) on the 4-point Likert scale. An I-CVI value of 0.78 or higher indicates excellent content validity (Polit et al., 2007). The average content validity was then calculated for the entire schedule (Scale Content Validity Index; S-CVI). S-CVI values between 0.80–0.89 are considered acceptable, and a value of 0.90 or above reflects excellent validity (Polit and Beck, 2006).

## 4 Results

### 4.1 ISPOR step 7: cognitive debriefing

#### 4.1.1 Phase A: focus groups

The healthcare professionals raised opinions on some phrasings in the questionnaire. Accordingly, six minor linguistic adjustments were made. The main concern was that, in English, certain feelings are expressed using multiple synonyms, whereas in Swedish they are typically conveyed with a single term. To align with this, three English synonyms were removed. One cultural adjustment was made. In Swedish, the word depressed refers to a clinical diagnosis, not a feeling, and was therefore replaced by a term that specifically relate to the feeling of being depressed.

#### 4.1.2 Phase B: interviews

Five of the patients interviewed had no specific comments or suggestions of changes whereas the remaining three patients (who had professions such as teacher and copywriter) suggested four minor linguistic adjustments; all of which were taken into account to make the questionnaire more comprehensible for a layperson.

#### 4.1.3 Phase C: survey regarding content validity

Sixteen out of the 18 questions were scored as having excellent Item Content Validity Index (I-CVI) of between 0.8 and 1 (Table 2). The remaining two questions had an I-CVI of 0.7, indicating a less good fit. The questions with lower I-CVI were questions “6. How happy, satisfied, or pleased have you been with your personal life?” and “11. Has your daily life been full of things that were interesting to you?.” The questionnaire as a whole had excellent content validity with a Scale Content Validity Index (S-CVI) of 0.91.

**Table 2:** Item and scale content validity indices (I-CVI, S-CVI) for the general well-being schedule.

Question GWB	Expert 1	Expert 2	Expert 3	Expert 4	Expert 5	Expert 6	Expert 7	Expert 8	Expert 9	Expert 10	Experts with √	I-CVI^*^
1. How have you been feeling in general?	√	√	√	√	√	√	√	√	√	√	10	1.00
2. Have you been bothered by nervousness or your nerves?	√	√	√	√	√	√	√	—	√	√	9	0.90
3. Have you been in firm control of your behavior, thoughts, emotions, or feelings?	√	√	√	√	√	—	√	√	√	√	9	0.90
4. Have you felt so sad, discourages, hopeless, or had so many problems that you wondered if anything was worthwhile?	√	√	√	√	√	—	√	√	√	√	9	0.90
5. Have you been under or felt you were under any strain, stress, or pressure?	√	√	√	√	√	√	√	√	√	√	10	1.00
6. How happy, satisfied, or pleased have you been with your personal life?	√	—	√	√	√	√	√	—	—	√	7	0.70
7. Have you had any reason to wonder if you were losing your mind, or losing control over the way you act, talk, think, feel, or of your memory?	√	√	√	√	√	√	√	—	√	√	9	0.90
8. Have you been anxious, worried, or upset?	√	√	√	√	√	√	√	√	√	√	10	1.00
9. Have you been waking up fresh and rested?	√	√	√	√	√	√	√	√	√	√	10	1.00
10. Have you been bothered by any illness, bodily disorder, pains, or fears about your health?	√	√	√	√	√	√	√	—	√	—	8	0.80
11. Has your daily life been full of things that were interesting to you?	√	—	√	√	√	√	√	—	√	—	7	0.70
12. Have you felt down hearted and blue?	√	√	√	√	√	√	√	√	√	√	10	1.00
13. Have you been feeling emotionally stable and sure of yourself?	√	√	√	√	√	√	√	—	√	√	9	0.90
14. Have you felt tired, worn out, used-up, or exhausted?	√	√	√	√	√	√	√	√	√	√	10	1.00
15. How concerned or worried about your health have you been?	√	√	√	√	√	√	√	—	√	√	9	0.90
16. How relaxed or tense have you been?	—	—	√	√	√	√	√	√	√	√	8	0.80
17. How much energy, PEP, and vitality have you felt?	√	√	√	√	√	√	√	√	√	√	10	1.00
18. How depressed or cheerful have you been?	√	√	√	√	√	√	√	√	√	√	10	1.00
											S-CVI^†^	0.91

Swedish translation and cultural adaptation as perceived by 10 healthcare professionals. √ shows the number of professionals that rated 3 = relevant or 4 = very relevant. ^*^Item Content validity Index (I-CVI): proportion of healthcare professionals giving ratings of 3 or 4. The I-CVI cut-off for excellent is ≥0.78. ^†^Scale Content Validity Index (S-CVI): Average of I-CVIs. The S-CVI cut-off for acceptability is ≥0.80–0.89 and is considered excellent at ≥0.90.

In response to the open-ended questions, addressing aspects related to relevance and operational use, the following three comments were made: (a) *I think all the questions can give a general picture of well-being. However, these types of questions are always a bit “rigid,” but it’s hard to broaden them without going overboard. (b) Good questions, they cover both negative and positive feelings/experiences. (c) Some questions could also be even more relevant to patients with tinnitus.* No adjustments were made based on these comments.

### 4.2 ISPOR step 8–10

Although the two questions with the lowest I-CVI were below the limit for “excellent” validity, we decided to retain both questions to avoid deviating from the original, validated English version. The final proofreading resulted in 9 minor grammatical corrections. The final translated Swedish version of the GWB, named *GWB: enkät om allmänt välbefinnande*, *svensk version*, can be downloaded from the Open Science Framework.^1^

## 5 Discussion

This project aimed to translate the General Well-Being Schedule (GWB) to develop a translated and culturally adapted Swedish version. To ensure high content validity, both linguistically and culturally, we strictly followed the ISPOR guidelines, including inviting both patients and healthcare professionals to attain a broad perspective of opinions. This method enabled the development of a linguistically and culturally adapted instrument, tailored to the Swedish context and easily understood by Swedish patients with sensory dysfunctions.

The content validity of the translated questionnaire was rated excellent with a Scale Content Validity Index (S-CVI) of 0.91. In total, 16 of the 18 questions were rated as having excellent validity, with an Item Content Validity Index (I-CVI) of between 0.8 and 1, indicating high validity for the individual questions. Four of the clinical experts rated all questions as relevant, and five rated 1 to 3 questions as less relevant. One clinician was an outlier, rating 7 questions as irrelevant. Nonetheless, the overall results demonstrate that the Swedish translation of the scale has a clear clinical relevance.

Two questions had a lower I-CVI (0.7). These addressed contentment with one’s private life and whether daily life was filled with things of interest. One of the questions (question 11) has previously been deemed as having low correlation with the other questions, and models excluding it showed increased fitness index (Nakayama et al., 2000). However, the same study also reported high reliability for the full version, including question 11, with a Cronbach’s alpha of 0.9. To experience well-being despite ill health, contentment with private life, relationships, and social engagement is crucial (Lamu and Olsen, 2018). We therefore regard it as essential to explore the patients’ contentment in these areas despite their lower rating. We further argue that it is important to not deviate too much from the original questionnaire, as well as to keep the same number of questions, as it has demonstrated high reliability and validity across populations (Poston et al., 1998; Taylor et al., 2003; Nakayama et al., 2000; Leonardson et al., 2003).

The healthcare professionals in the focus groups expressed concerns about certain adjectives used in the form, stating that they felt “American” and unnatural in Swedish. These, along with some synonyms, were changed to make the language more suitable for the cultural setting. The patients interviewed did not express as many opinions as the healthcare professionals. One explanation may be that the phrasing had already been improved based on the input from the healthcare professionals by the time the patients reviewed the questionnaire.

### 5.1 Implications for clinical practice

The GWB is a validated instrument considered suitable for investigating general well-being in various patient groups and populations (Poston et al., 1998; Taylor et al., 2003; Nakayama et al., 2000; Leonardson et al., 2003). Until now, there has not been a Swedish translation of the instrument adapted to a Swedish setting and evaluated by Swedish patients or professionals. The general well-being, including the patients’ private life, is of importance for patients suffering from different conditions in their rehabilitation (Lamu and Olsen, 2018), and it is therefore crucial to focus not only on disease burden or symptoms severity when evaluating well-being. A Swedish version of the GWB could provide new insights into the well-being of patients whose primary issues are not related to pain or motor function. This allows for improved treatment, not only of the condition itself but also of its psychological aspects. It also provides better insight into what it is like living with sensory disorders in Sweden. Likely, the questionnaire could also be suitable for other Swedish-speaking patient groups; however, this would need to be confirmed separately.

### 5.2 Strengths and limitations

An important strength of the present study lies in the use of a methodology aligned with the ISPOR Guidelines (Wild et al., 2005). By using the ISPOR guidelines when translating and culturally adapting the questionnaire to a Swedish healthcare setting, we have ensured a structured and validated approach to the work. Both patients and healthcare professionals have been involved in the cognitive debriefing process, providing a wide range of perspectives on the questionnaire, ensuring that the questions are understood correctly and fit into the cultural context. Further analyses of the questionnaire itself, including reliability and construct validity testing, have already been performed through several independent studies (Poston et al., 1998; Taylor et al., 2003; Nakayama et al., 2000; Leonardson et al., 2003). Future studies may evaluate the psychometric properties of the Swedish version according to COSMIN recommendations, including structural validity, internal consistency, reliability, and construct validity through hypothesis testing (Terwee et al., 2018).

When choosing participants for the patient interviews, a convenience sample of patients who had recently visited the hospital was used. Although the study aimed to include patients across a broad age range, no participants were under 30 or over 70 years old. Nevertheless, the age distribution of the sample accurately reflects the typical patient population at the clinic. Similarly, there was a majority of female patients in the Phase B interviews, as female patients are overrepresented in terms of seeking medical care for sensory dysfunction. Furthermore, a strength of the study is the lack of drop-out, as all patients who were asked to participate accepted the opportunity. It is, however, possible that a different result would have occurred had we chosen a different sampling method. All patients were recruited at the same hospital and lived in Stockholm. It is possible that asking patients from geographically different or more rural parts of the country could provide different results. The small sample size of patients and professionals involved in the translation process could be regarded as a limitation in terms of generalizability and a study on a larger patient population could be beneficial. However, the ISPOR guidelines recommend involving a group of 5–8 respondents and emphasize the importance of a representative sample (Wild et al., 2005). In the present study 8 patients and 16 healthcare professionals were involved, and the sample of patients well represents the typical patient population in terms of age and gender.

## 6 Conclusion

The Swedish version of the General Well-Being Schedule was successfully translated and culturally adapted in strict accordance with ISPOR guidelines. The results indicate good content validity and comprehensibility.

## Data Availability

The original contributions presented in the study are included in the article, further inquiries can be directed to the corresponding author/s.

## Footnotes

1
OSF|GWBSwedish.pdf
